# Supplementary material for: Ocean Acidification Affects the Response of the Coastal Coccolithophore Pleurochrysis carterae to Irradiance
Source: Biology (Basel). 2023 Sep 18;12(9):1249. doi: 10.3390/biology12091249 (PMC10525560; doi:10.3390/biology12091249)

Supporting Information.

Table S1. List of the physiological responses of coccolithophores *E. huxleyi* and *G. oceanica* under different CO_2 and irradiance treatments, extracted for previously published studies (Jin et al., 2017; Zhang et al., 2015).

Strain	Treatment		Parameters		
	pCO_2 (ppm)	Irradiance (μmol photons $m^{-2} \cdot s^{-1}$)	μ_{max} (d^{-1})	POC $Prod_{max}$ (pg $cell^{-1}$ d^{-1})	PIC $Prod_{max}$ (pg $cell^{-1}$ d^{-1})
E. huxleyi	~ 400	54	0.60	8.50	5.10
		83	0.65	10.50	5.00
		141	0.64	10.20	5.40
		257	0.68	12.30	5.90
		456	0.57	10.70	3.10
	~ 1000	54	0.59	10.00	2.00
		83	0.72	11.30	3.00
		141	0.77	12.40	4.00
		257	0.8	13.80	4.10
		456	0.85	18.00	3.20
G. oceanica	~ 500	50	0.5	4.09	5.340
		100	0.72	12.27	17.14
		200	1.09	21.27	36.84
		400	1.19	40.28	55.43
		600	1.21	39.43	63.75
		800	1.26	37.13	61.46
	~ 1000	50	0.52	3.68	3.62
		100	0.78	13.92	13.47
		200	0.9	23.24	17.27
		400	0.95	27.23	17.77
		600	0.89	18.83	20.78
		800	0.88	17.50	20.55

Data extracted from Jin et.al (2017) for *E. huxleyi* and from Zhang et.al (2015) for *G. oceanica*. All data are mean values of parallel samples. *E. huxleyi* was maintained in artificial seawater enriched with Aquil medium at 20°C. *G. oceanica* was grown in artificial seawater (ASW) at 20 ℃ with a 16: 8 h light: dark photo-period in a RUMED Light Thermostat.

Figure S1: Comparison of the effects of OA and irradiance on the physiology of coccolithophores *P. carterae, E. huxleyi* and *G. oceanica*. (A) Growth rate vs. irradiance; (B) POC production rate *vs.* irradiance;(C) PIC production rate *vs.* irradiance. The growth rate *vs.* irradiance curves was fitted using a model of Eilers and Peters (1988). The POC and PIC production rate *vs.* irradiance curves (P-I curves) were fitted to the Steele model (1962), while the POC production rate *vs.* irradiance curves of *E. huxleyi* was fitted to the model of Eilers and Peters (1988).

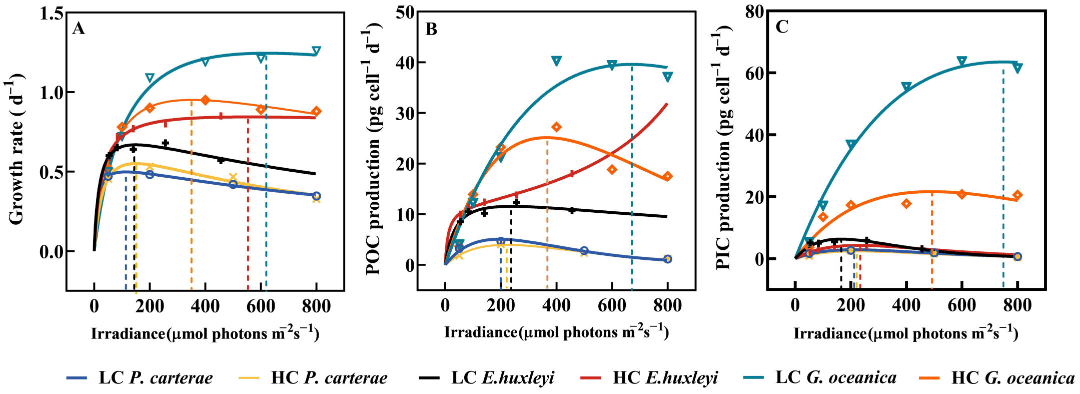

Data extracted from Jin et.al (2017) for *E. huxleyi* and from Zhang et.al (2015) for *G. oceanica*. (LC means low CO_2 concentration, HC means high CO_2 concentration.)

Figure S2. The box plot of cell sizes of the coccolithophore of *P. carterae* under different CO_2 and irradiance treatments. Center lines show the medians, box limits indicate the 5th and 75th percentiles and outliers are represented by dots. (LC means low CO_2 concentration, HC means high CO_2 concentration.)

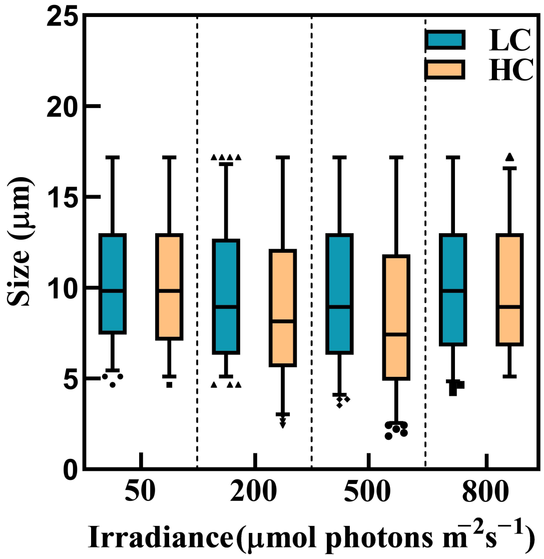

Supplement: Supplementary file 1 [file biology-12-01249-s001.zip › Supporting Information.pdf]
